# Network-Based Approaches for Disease-Gene Association Prediction Using Protein-Protein Interaction Networks

**DOI:** 10.3390/ijms23137411

**Published:** 2022-07-03

**Authors:** Yoonbee Kim, Jong-Hoon Park, Young-Rae Cho

**Affiliations:** 1 Division of Software, Yonsei University Mirae Campus, Wonju-si 26493, Gangwon-do, Korea; yoonbee7@yonsei.ac.kr (Y.K.); jongh36@yonsei.ac.kr (J.-H.P.); 2 Division of Digital Healthcare, Yonsei University Mirae Campus, Wonju-si 26493, Gangwon-do, Korea

**Keywords:** disease-gene associations, disease gene prioritization, protein-protein interaction networks, disease networks, heterogeneous networks

## Abstract

Genome-wide association studies (GWAS) can be used to infer genome intervals that are involved in genetic diseases. However, investigating a large number of putative mutations for GWAS is resource- and time-intensive. Network-based computational approaches are being used for efficient disease-gene association prediction. Network-based methods are based on the underlying assumption that the genes causing the same diseases are located close to each other in a molecular network, such as a protein-protein interaction (PPI) network. In this survey, we provide an overview of network-based disease-gene association prediction methods based on three categories: graph-theoretic algorithms, machine learning algorithms, and an integration of these two. We experimented with six selected methods to compare their prediction performance using a heterogeneous network constructed by combining a genome-wide weighted PPI network, an ontology-based disease network, and disease-gene associations. The experiment was conducted in two different settings according to the presence and absence of known disease-associated genes. The results revealed that HerGePred, an integrative method, outperformed in the presence of known disease-associated genes, whereas PRINCE, which adopted a network propagation algorithm, was the most competitive in the absence of known disease-associated genes. Overall, the results demonstrated that the integrative methods performed better than the methods using graph-theory only, and the methods using a heterogeneous network performed better than those using a homogeneous PPI network only.

## 1. Introduction

Identification of genes causing diseases is a primary goal in human health research for accurate disease diagnosis, treatment, and prevention [1,2]. In the process of cloning and dividing genes, structural changes can occur in a gene that can transform biological processes and cause diseases. Existing methods of genome-wide association studies (GWAS) infer genome intervals that are involved in genetic diseases [3,4,5]. However, because GWAS are used to investigate millions of putative genetic mutations, collecting candidate genes is a time-consuming and expensive task. To resolve such issues of GWAS, recent computational approaches have focused on the systematic analysis of molecular networks to predict associations between diseases and genes. These network-based methods have become an effective strategy to complement GWAS.

Numerous network-based approaches have recently been proposed for disease-gene association prediction. In this article, we provide a broad overview of the network-based methods. The underlying assumption of these methods is that phenotypically similar diseases are caused by functionally related genes which are located close to each other in molecular networks, such as protein-protein interaction (PPI) networks, co-expression networks, and gene regulatory networks [6,7,8,9,10]. Among these molecular networks, PPI networks are the most frequently used because proteins that interact with each other perform common biological functions. Moreover, proteins with similar functional roles have common topological features in the PPI network, such as the degree and centrality of nodes, and the pathways elucidating disease mechanisms are typically represented as strongly connected paths in the PPI network.

However, it is widely known that experimentally determined PPI datasets include numerous false positives. In addition, the PPI datasets that are obtained in a genomic scale can be biased toward highly studied proteins. Therefore, the genome-wide PPIs from many open-source databases have been curated using domain knowledge such as gene expression and gene ontology (GO). For our experiments in this article, we use a genome-wide PPI network that is weighted by GO and its annotation data [11]. GO provides a standardized structure for biological terms by linking them based on the relationships between their meanings. GO also provides annotations of genes and gene products for these terms. The functional relationships among interacting proteins can thus be evaluated using GO structures and annotation data to curate PPI data, as mentioned previously herein.

We can divide network-based approaches for disease-gene association prediction into three groups as follows: methods using graph-theoretic algorithms, such as random walks, network propagation, and path search; those using machine learning algorithms, such as deep learning; and those integrating graph theory and machine learning techniques. Because previous methods in these three categories have been evaluated using different data sources and different experimental conditions, it has been difficult to rationally compare their prediction performance. In this survey, we define the disease-gene association prediction task and provide an overview of the state-of-the-art network-based approaches. In addition, we experimented with several selected methods to compare their prediction performance using a uniform dataset that includes a PPI network, a disease network and known disease-gene associations. This experiment was conducted in two different settings according to the presence and absence of known disease-associated genes.

### Disease-Gene Association Prediction

Predicting disease-gene associations, also called disease gene prioritization, is a task to identify all genes that are involved in a disease. Network-based computational methods predict associations by measuring the likelihood of genes being linked to a disease in a network. The networks used in these methods can be categorized into homogeneous, heterogeneous, and multiplex networks according to the types of nodes and edges [12].

First, a homogeneous network consists of a single type of node and edge. This can be denoted as G=(V,E), where *V* is a set of nodes and *E* is a set of edges. As a typical example, a PPI network is constructed with proteins as nodes and PPIs as edges. Second, a heterogeneous network is created by integrating two or more homogeneous networks and linking them together. For example, in a disease-gene heterogeneous network G=(V,E), *V* consists of two different sets of nodes of $V=\{V_{diseases}\cup V_{genes}\}$, and *E* consists of three different sets of edges, such as disease similarities, PPIs, and disease-gene associations, represented as $E=\{E_{diseases}\cup E_{genes}\cup E_{associations}\}$. Finally, a multiplex network consists of a single type of node and several types of edges. For example, a multiplex network of proteins G=(V,E) can be constructed using *V* as a set of proteins and *E* consisting of PPIs, pathways, and co-expressions.

Disease-gene association prediction methods can return a list of candidate genes for each disease. Methods using a disease-gene heterogeneous network as input measure the likelihood of genes being linked to the query disease and list the candidates. However, methods using a PPI homogeneous network as input measure the similarity between genes and each known gene associated to the query disease, called a seed gene, to select candidates. Therefore, in this case, at least one disease-associated gene should be known.

## 2. Survey of Network-Based Disease-Gene Association Prediction Methods

Existing network-based methods for disease-gene association prediction are listed in Table 1. This table includes data sources that are used for each method, the network type that each method can accept, and key techniques that are applied to each method. The methods used disease data from OMIM [13] or HPO [14]; PPI data from HPRD [15], BIND [16], BioGrid [17], IntAct [18], STRING [19], ConsensusPathDB [20] or Interactome [21]; disease-gene association information from OMIM, HPO, DisGeNet [22], or CTD [23]; gene-gene similarity information from GO [11] or co-expression databases; and disease-disease similarity information from HPO or MimMiner [24]. We survey the network-based methods in three categories in this section.

### 2.1. Methods Using Graph-Theoretic Algorithms

#### 2.1.1. RWR

While typical network-based disease-gene association prediction methods prioritize genes using only a local part of the network, such as direct interactions and shortest paths, RWR (Random Walk with Restart) [25] adopts random walk to prioritize candidate genes in a global network. Random walk is the process of a walker moving in random directions in a graph. That is, a walker randomly selects and moves to its neighboring nodes. In a weighted graph, the probability of selecting a neighboring node reflects the weight of the edge between the current and neighboring nodes.

RWR provides the restart probability to a random walk for every step *t*. The walker then returns to the starting node with a probability of *r* at every step or moves randomly to a neighboring node with a probability of 1−r, as shown in the following equation:

(1) $$p^{t+1}=\left(1-r\right)W^{\prime }p^{t}+rp^{0}$$

where $p^{t}$ is the probability that the walker stands in each node at *t*; $p^{0}$ is the initial probability vector, with all start nodes having equivalent values and the rest consisting of zero; and $W^{\prime }$ is a transition matrix, which is a transposed matrix of the column-normalized adjacency matrix of the graph. If this step of the random walk is sufficiently repeated, then *p* becomes the steady-state probability vector $p^{\infty }$ which is a state such that the difference between $p^{t}$ and $p^{t+1}$ was less than $10^{-6}$ in the L1 norm.

#### 2.1.2. RWRH

Because functionally similar genes can cause similar diseases, candidate genes can be prioritized using other genetic and phenotypic data. In this sense, RWR was extended to RWRH (Random Walk with Restart on Heterogeneous network) [26] using a heterogeneous network. That is, the gene network and phenotype network are merged into a heterogeneous network by gene-phenotype associations, which represents a bipartite graph. In RWRH, the transition matrix of the heterogeneous network is redefined as a combined form of the transition matrices of individual networks. In addition, the jumping probability is given to allow a walker in the gene network to jump to the phenotype network or vice versa via gene-phenotype associations. The initial probability vector assigns different weights to seed nodes.

#### 2.1.3. PRINCE

PRINCE (PRIoritizatioN and Complex Elucidation) [27] adopts a propagation algorithm that uses the global topology of a heterogeneous network to prioritize disease genes and infers protein complexes. The underlying principle of this method is that prior information is propagated through the associations of diseases similar to the query disease. The overall process of PRINCE is very similar to that of RWR, but its transition matrix contains information regarding the node where the flow of prior information arrives, as well as the node where the flow of information is sent.

#### 2.1.4. DADA

Erten et al. [28] demonstrated that the performance of global prioritization methods, such as random walk and network propagation, is highly biased toward the degree of candidate genes. They proposed a strategy of statistical adjustment that adjusts the degree bias to improve the performance of genes in low degrees. They used the degree distribution of PPI networks based on three reference models, the degree of seed nodes, degree of candidates, and likelihood ratio using eigenvector centrality. These models can be used to evaluate the statistical importance of the links between candidate and disease genes. However, these models identify lowly-connected genes precisely but do not work well for highly-connected genes, suggesting a method that combines raw scores and statistically adjusted scores. Therefore, a uniform prioritization method, named DADA, was created to prioritize candidate genes using raw scores for genes with high connections and adjusted scores for genes with low connections.

#### 2.1.5. RWR-MH

To address the typical noise issues of biological data, Valdeolivas et al. [29] suggested the integration of multiple sources. However, aggregating them might ignore the features and topologies of each network. Therefore, they introduced a multiplex network that shares nodes with different types of edges. RWR-MH (RWR to Multiplex-Heterogeneous networks) [29] uses a combination of a multiplex network and a heterogeneous network as an extension of RWRH. After composing a single multiplex network of genes by integrating PPIs, pathways, and co-expressions at each layer, the multiplex network was connected to a disease network by associations. In this network, a walker can move to the same node in another layer, to a neighboring node in the same layer, or to a different network through an association.

#### 2.1.6. PhenoRank

PhenoRank [30] uses PPIs and phenotypes from evolutionarily distant species, such as humans and mice, to prioritize disease-associated genes. Similarities between phenotypes measured using ontologies and annotations can be biased because of the numbers of phenotype terms and annotating genes, which reflect how well these entities are studied. To avoid this problem, PhenoRank computes gene scores based on the sum of the phenotypic similarities with the associated diseases and mutants of orthologous mouse genes. The gene scores are propagated through the PPI network such that the genes interacting with high-scoring genes also score high. The gene score generated for the query disease is compared with the gene score generated for the simulated set of phenotype terms, and the *p*-value is calculated for each gene. Candidate genes are prioritized by these *p*-values, which represent the probability of a gene score at least as great as that observed.

#### 2.1.7. NetCore

Barel and Herwig [31] proposed NetCore, a method that uses network propagation with node coreness to predict phenotype-gene associations and identify modules. Node coreness is a topological feature that reflects whether a node belongs to a densely connected part of the network or its periphery. This can be calculated using the k-shell decomposition algorithm. For network propagation, they used a permutation test, which is network randomization using the double-edge swap algorithm, and re-ranked the genes by *p*-values with the same significance level. The authors recommended the use of 100 permutations. They also normalized the adjacency matrix of the PPI network based on node degree, core, the difference between the node degree and core, and the ratio between the node degree and core.

#### 2.1.8. PRYNT

PRYNT [32] applies a combination of the shortest-path search and random walk to a PPI network to prioritize disease genes. The PPI network from the STRING database is contextualized by adding the deregulated proteins regardless of their confidence level and by grouping the proteins within the cliques. The rank of proteins is obtained by multiplying the ranks from the shortest-path search algorithm and random walk. They demonstrated that this combination strategy performs better than direct ranking.

### 2.2. Methods Using Machine Learning Algorithms

#### 2.2.1. CIPHER

CIPHER (Correlating protein Interaction network and PHEnotype network to pRedict disease genes) [33] observed that genes causing the same or similar diseases are often located close to each other in a PPI network. A similarity profile is composed of the similarities between the query phenotype and other phenotypes, and a closeness profile is calculated using the topological distance between the candidate gene and all known disease genes. The relationship between the two profiles is quantified using a concordance score and modeled by regression. CIPHER includes two different types, CIPHER-SP and CIPHER-DN, depending on whether the shortest paths or direct neighbors are used when calculating gene closeness.

#### 2.2.2. CrossRankStar

Ni et al. [34] pointed out that previous gene prioritization methods tend to assume that all diseases share the same molecular network, and they use a single network to rank disease-related genes. However, a disease can occur in different tissues, and the molecular networks in these tissues are usually different. They thus proposed the construction of tissue-specific networks, named NoN (network of networks) and NoSN (network of star networks). In NoN, each disease in the disease network has its own tissue-specific molecular network. NoN can be expanded to NoSN, which has a tissue-specific network for each disease consisting of a central network and auxiliary networks. They developed prioritization methods called CrossRank and CrossRankStar, which can be applied to NoN and NoSN, respectively. These methods repeatedly update the ranking score vector of genes in an objective function based on three criteria: within-network smoothness, within-network seed preference, and cross-network consistency that is optional. The objective function is finally minimized by gradient descent.

#### 2.2.3. pBRIT

Kumar et al. [35] categorized several data sources such as PubMed abstracts, GO, Human Phenotype Ontology (HPO), protein sequence similarities, pathways, PPIs, and gene associations into functional and phenotypic annotations. Their method, pBRIT (Prioritization using Bayesian Ridge regression and Information Theoretic model), uses a text-mining technique, such as TF-IDF, to provide a static weight for each pair between a gene and a feature in the sparse annotation matrices. For each entry of the matrices, TF-IDF multiplies the frequency of each feature in a gene by the inverse frequency of the feature in all genes. If the feature of a gene has a high frequency and the feature of all genes has a low frequency, then the feature has a high weight with the gene. The gene-gene proximity profiles are calculated as the cosine similarity of TF-IDF and SVD transformed from the annotation matrices. According to the hypothesis that genes causing similar diseases share functional and phenotypic characteristics, functional and phenotypic annotations can be formulated by regression. Because regression is affected by model uncertainty, the uncertainty of annotations is modeled by a Bayesian approach while learning the linear mapping between functional and phenotypic annotation sources.

#### 2.2.4. Scuba

Scuba [36] is a scalable kernel-based method for disease gene prioritization. Among the multiple kernel learning (MKL) algorithms for data integration, Scuba adopted EasyMKL [39], which computes the optimized kernel without being affected by the number of kernels, with linear time complexity. It maximizes the distance between positive and negative examples and optimizes the margin distribution. However, for disease gene prioritization, the following difficulties are commonly encountered. First, the number of known disease genes is extremely small compared to the number of candidates. Second, the negative examples are not certain. To solve these problems of an unbalanced setting, Scuba expanded EasyMKL by dividing the kernel into positive and unlabeled sub-kernels.

### 2.3. Integrated Methods of Graph Theory and Machine Learning

#### 2.3.1. IDLP

Zhang et al. [37] attempted to tackle the issue that numerous false-positive interactions exist in the PPI data set. Considering the PPI network as a variable, noise was reduced by optimizing the loss function. They proposed IDLP (Improved Dual Label Propagation) which optimizes the following objective function

(2) $$L\left(Y,S_{1},S_{2}\right)=L_{1}\left(Y,S_{1}\right)+L_{2}\left(Y,S_{2}\right)$$

where *Y* is a disease-gene association matrix to be learned, and $S_{1}$ and $S_{2}$ are the weighted PPI network and weighted disease network, respectively. In this formula, $L_{1}$ is the objective function when label propagation is performed for all phenotypes in the PPI network assuming that the PPI network contains noise. $L_{2}$ is the objective function when label propagation is performed for all genes in the disease network assuming that the disease network contains noise.

#### 2.3.2. HerGePred

Yang et al. [38] integrated disease-gene associations, disease-symptom associations, PPIs, and GO term annotations into a heterogeneous disease-gene network (HDGN) and proposed HerGePred by applying network embedding representation algorithms such as node2vec [40] and LINE [41] to obtain low-dimensional vector representation (LVR) in HDGN. Subsequently, they proposed LVR-based similarity prediction (LVRSim) and random walk with restart based on a reconstructed heterogeneous disease-gene network (RW-RDGN). In LVRSim, because LVR can contain the local and global structure information of the network, disease-gene similarity can be computed using LVR-based cosine similarity. RW-RDGN reconstructs a heterogeneous network using new disease and gene networks. Finally, the RWR algorithm is applied to the reconstructed heterogeneous network to prioritize the candidate genes.

## 3. Experiments

### 3.1. Experimental Data

#### 3.1.1. Gene Networks

For our experiment, human PPI data were acquired from BioGRID [17] and filtered for physical interactions. The self-loops were eliminated. The interacting proteins were paired with the genes that were produced, maintained, and treated as gene-gene interactions.

For weighting interactions, we quantified functional similarity between interacting genes based on semantic similarity using GO [11]. GO is structured based on the parent-child relationships of its terms and provides comprehensive annotations of molecular products based on published evidence. In GO, we extracted terms and their relationships in the domains of molecular function and biological process, excluding cellular components. In addition, to ensure the quality of the annotation data, we removed annotations with the evidence code of IEA. If any genes from the PPI data were not included in the GO annotations, their interactions were eliminated.

The functional similarity of interacting gene pairs was computed by integrating term-based and annotation-based semantic similarity measures [42,43,44]. The semantic similarity between two GO terms is defined as follows:

(3) $$sim\left(C_{1},C_{2}\right)=\frac{\sum_{C_{i}\in A_{t}\left(C_{1}\right)\cap A_{t}\left(C_{2}\right)}\log P\left(C_{i}\right)}{\sum_{C_{j}\in A_{t}\left(C_{1}\right)\cup A_{t}\left(C_{2}\right)}\log P\left(C_{j}\right)}$$

where $C_{1}$ and $C_{2}$ are the GO terms annotating genes $g_{1}$ and $g_{2}$, $A_{t}\left(C_{1}\right)$ and $A_{t}\left(C_{2}\right)$ are the sets of the ancestral terms of $C_{1}$ and $C_{2}$, respectively, in the ontology. $P(C_{i})$ is the probability of being annotated to term $C_{i}$, and $-\log P(C_{i})$ indicates the information content of $C_{i}$. The semantic similarities of all term pairs annotating $g_{1}$ and $g_{2}$ are aggregated to the functional similarity between $g_{1}$ and $g_{2}$ by best-match averaging, which returns the average of the best matching semantic similarity scores for each term as follows:

(4) $$sim\left(g_{1},g_{2}\right)=\frac{\sum_{i}\max_{j}sim\left(C_{i},C_{j}\right)+\sum_{j}\max_{i}sim\left(C_{i},C_{j}\right)}{|C_{i}\left|+\right|C_{j}|}$$

where $|C_{i}|$ and $|C_{j}|$ are the numbers of GO terms annotated as $g_{1}$ and $g_{2}$, respectively. This process creates a gene network as an undirected weighted graph, which was found to comprise 14,663 genes as nodes and 258,476 edges weighted by Equation (4).

#### 3.1.2. Disease Networks

A disease network for our experiment was constructed using Human Phenotype Ontology (HPO) [14]. We extracted all sub-terms of Phenotypic Abnormality in HPO, which indicate abnormal phenotypes caused by any disease or disorder. The diseases from OMIM [13], OrphaNet [45] and DECIPHER [46] were annotated to the terms in HPO. However, in our experiment, the annotations of diseases from OMIM were only used because the diseases were linked to genes based on disease-gene associations provided by OMIM.

Similarities between all possible pairs of diseases were measured using the same semantic similarity method as that used for weighting the gene network. The similarities between HPO terms were computed using Equation (3) where $C_{1}$ and $C_{2}$ are HPO terms annotating diseases $g_{1}$ and $g_{2}$. The similarity between the diseases $g_{1}$ and $g_{2}$ were computed using Equation (4). A disease network was formed by adding the weighted edges for the disease pairs with similarities greater than 0.1. This was found to comprise 6465 diseases as nodes and 4,354,956 edges, with a density of 20% approximately.

#### 3.1.3. Disease-Gene Associations

Disease-gene associations were acquired from the morbid map of the OMIM database [13]. OMIM provides information regarding the relationship between phenotypes and genotypes. For our experiment, associations with genes or diseases that do not occur in our gene network or disease network were removed. Finally, we obtained 5024 disease-gene associations, which created a heterogeneous network including 3258 genes and 4506 diseases. The statistics of our experimental dataset including the gene network, the disease network, and disease-gene associations are summarized in Table 2.

### 3.2. Experimental Settings

For this experiment, we randomly extracted two samples consisting of 100 diseases and their associations. Sample-1 was extracted only for diseases with more than one disease-gene association, and sample-2 was extracted regardless of the number of disease-gene associations including the case of diseases with a single disease-associated gene. Sample-1 consisted of 100 diseases, 357 genes and 431 associations whereas sample-2 consisted of 100 diseases, 166 genes and 175 associations. In sample-2, only 14 diseases had two or more associations with genes. Using these samples, leave-one-out cross-validation was performed to evaluate the accuracy of disease-gene association prediction. In other words, we iteratively used each disease and its association(s) in the samples as test data and computed the area under the curve (AUC). The AUC is generally considered the most effective means of measuring predictive power. We also showed recall, which is the ratio of correctly predicted associations (i.e., true positives) to all actual associations, for prediction accuracy.

Our experiment was performed in two different settings as follows: (setting-1) prediction in case that known disease-associated genes of the disease of interest exist and (setting-2) prediction in case that known disease-associated genes of the disease of interest do not exist. In setting-1, we removed only one association of each disease from sample-1 to predict the removed association. However, in setting-2, we removed all associations of each disease from sample-2 to predict any of them. In setting-1, each disease-gene association was in a query, whereas each disease was in a query in setting-2. Both settings returned the priority of genes for the disease of interest.

The results from these two settings were evaluated in two ways. First, we computed AUC and recall based on the ranks of the predicted genes for each sampled disease. Second, we computed them based on the prediction scores of genes across all sampled diseases. Further details are presented in the following sections.

## 4. Results

### 4.1. Prediction with Known Disease-Associated Genes

For the prediction accuracy comparison, we selected six previous methods, RWR, PRINCE, DADA, IDLP, HerGePred, and NetCore. The selected methods were executed in two different settings, as previously described. Our first experiment was to predict additional disease-gene associations when at least one disease gene was known. In other words, because each disease in sample-1 was associated with multiple genes, only one association with the disease became the test data and the rest became the training data.

#### 4.1.1. Evaluation by Ranks

First, we ranked the genes associated with each disease from sample-1. AUC and recall were computed using the prediction results with ranks higher than the rank parameter value. We tested with the rank parameter *r* at 100, 500, 1000, 5000, and 10,000. This indicates that we considered the top *r* predicted genes for each disease-gene association to measure the prediction accuracy. If there were *n* associations, the AUC and recall were computed based on the results of the total r×n cases. If the gene with a rank higher than *r* for each association was correctly predicted, it was considered a true positive. If not, it was considered a false positive.

The AUC and recall values are listed in Table 3 and Table 4, respectively. These results indicated that HerGePred performed best in the presence of known disease-associated genes. RWR, DADA, and NetCore tended to predict associations well when *r* was low. From this result, we can conclude that network propagation, which is a common technique applied by HerGePred, RWR, DADA and NetCore, is effective in increasing prediction accuracy.

**Figure 1:**
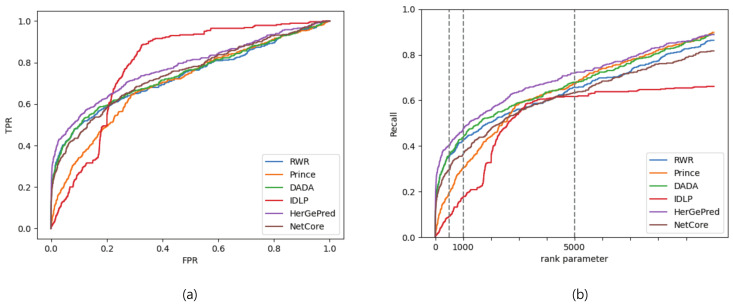
ROC (**a**) and recall (**b**) curves evaluated by gene ranks for disease-gene association prediction with known disease-associated genes.

Figure 1 shows the ROC and recall curves. The recall curve was created by varying *r* from 1 to 10,000, whereas the ROC result was achieved with a threshold *r* of 10,000. In other words, for the ROC curve, true positives and false positives above the threshold were only considered. These two plots indicate that HerGePred had the best predictive performance. Interestingly, the prediction accuracy of IDLP increased significantly when *r* was approximately 1800 as shown in Figure 1b.

#### 4.1.2. Evaluation by Prediction Scores

In the evaluation by ranking, even if genes are predicted in the same rank for different diseases, their prediction scores can be significantly different. To address this problem, we evaluated the prioritization results by aligning the genes in decreasing order of their prediction scores, regardless of the diseases associated with them. Similar to the rank parameter, we used the score parameter, which indicates the total number of predicted associations for evaluation.

To compare this with the results of the previous evaluation method based on ranks in Table 3 and Table 4, we set the number of predicted associations with all diseases using the score parameter *s* as r×n, where *r* is the rank parameter and *n* is the number of disease-gene associations. Because sample-1 contained 431 associations with all 100 diseases, *n* was 431 and the rank parameter *r* was set as 100, 500, 1000, 5000, and 10,000. If the gene with a rank higher than r×n across all sampled diseases was correctly predicted, it was considered a true positive. If not, it was considered a false positive.

**Figure 2:**
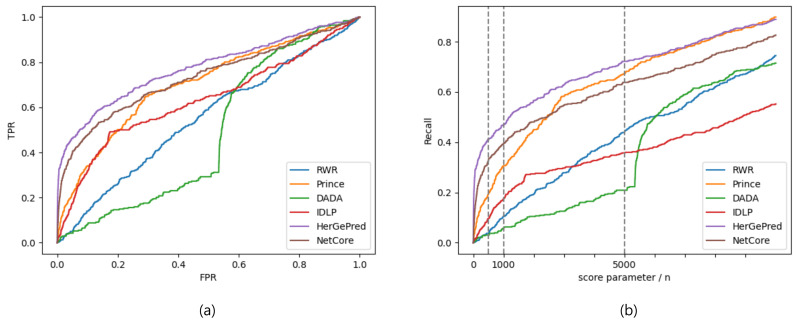
ROC (**a**) and recall (**b**) curves evaluated by prediction scores for disease-gene association prediction with known disease-associated genes.

The AUC and recall values are shown in Table 5 and Table 6, respectively. Similar to the previous results in Table 3 and Table 4, HerGePred had the best predictive performance, and NetCore and PRINCE showed relatively good performance. In Figure 2, the ROC and recall curves confirmed that HerGePred performed the best. The results showed that DADA had a sudden increase in prediction accuracy when *r* was slightly above 5000 as shown in Figure 2b. DADA typically scores in two different ways; one is achieved by random walks and the other is computed statistically. This could lead to inconsistencies in successful predictions.

### 4.2. Prediction without Known Disease-Associated Genes

To determine how well each selected method predicts disease-gene associations without any known disease genes, we removed all associations of each disease from sample-2 and predicted any associations that were removed. However, this setting cannot be implemented with RWR, DADA, and NetCore because these methods use a PPI network only and thus should preselect the seed genes from the PPI network with known associations. In contrast, because PRINCE, IDLP, and HerGePred take a disease-gene heterogeneous network as input, they can predict the genes associated with a disease that is not linked to the PPI network. Therefore, we applied this setting to PRINCE, IDLP, and HerGePred only.

#### 4.2.1. Evaluation by Ranks

Similar to that for setting-1 in Section 4.1, we used the ranking of the genes associated with each disease from sample-2. For this experiment, the same values of the rank parameter *r* were used as 100, 500, 1000, 5000 and 10,000. If the gene with a rank higher than *r* for each disease was correctly predicted, it was considered a true positive. If not, it was considered a false positive.

**Figure 3:**
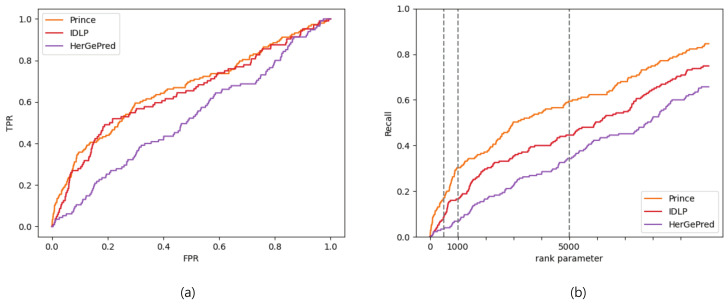
ROC (**a**) and recall (**b**) curves evaluated by gene ranks for disease-gene association prediction without known disease-associated genes.

The AUC and recall values are listed in Table 7 and Table 8, respectively. Unlike the results in Table 3 and Table 4, PRINCE had the best predictive performance in the absence of known disease genes, and HerGePred, which was the best in Table 3 and Table 4, was the least predictive among the three methods in this experiment. These results were also observed precisely in the ROC and recall curves as shown in Figure 3.

#### 4.2.2. Evaluation by Prediction Scores

The prediction results without any known disease genes were also evaluated using the prediction scores of the genes that were associated with all diseases in sample-2. To compare the results with those in Table 7 and Table 8, we set the score parameter *s* to r×m where *r* is the rank parameter and *m* is the number of sampled diseases which was 100. The same values of *r* were used as performed previously.

**Figure 4:**
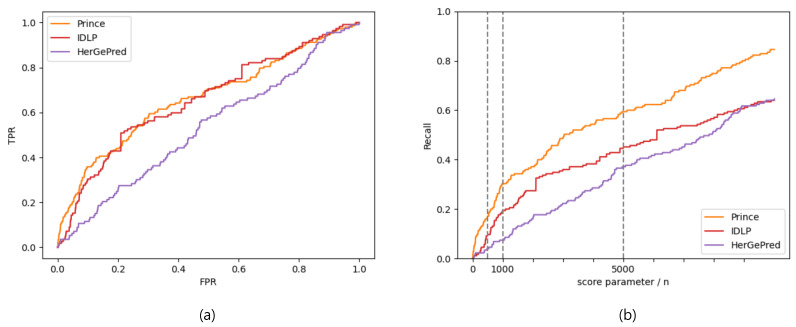
ROC (**a**) and recall (**b**) curves evaluated by prediction scores for disease-gene association prediction without known disease-associated genes.

The AUC and Recall values are shown in Table 9 and Table 10, respectively. The overall results were similar to those in Table 7 and Table 8. PRINCE had the highest prediction accuracy, where HerGePred had the lowest accuracy. These results were also demonstrated by the ROC and recall curves in Figure 4.

### 4.3. Degree Effect in Association Prediction

**Figure 5:**
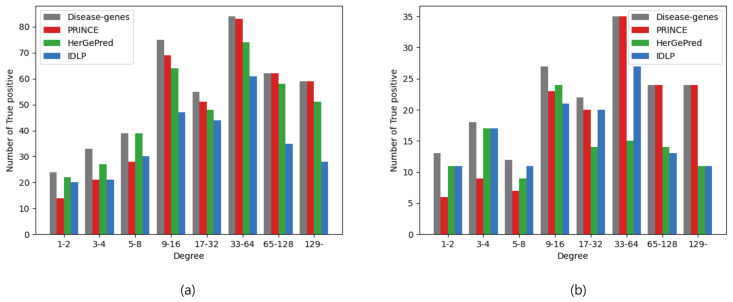
Numbers of disease-associated genes (i.e., ground-truth) and true positives with each method according to their degree in the PPI network in prediction with known disease genes from sample-1 (**a**) and without known disease genes from sample-2 (**b**).

We next tested whether the predictive performance of each method was affected by the degree of disease-related genes in the PPI network. It has been validated that high-degree proteins in a PPI network generally play significant roles as the functional cores in modules. In Figure 5, the grey bar represents the number of disease genes with respect to their degree in the PPI network, which indicates the ground truth. The other bars represent the number of true positives, which indicate the disease genes that each method successfully predicted. Figure 5a shows the prediction results with known disease genes using sample-1, and Figure 5b shows the prediction results without known disease genes using sample-2. In these Figures, we discretized the degrees in the range between $2^{i-1}+1$ and $2^{i}$ where *i* is a positive integer.

PRINCE, which applies simple network propagation, had more true positives with genes in higher degrees. However, it did not accurately predict genes in lower degrees. The prediction accuracy of PRINCE had a positive relationship with the degrees of genes. This pattern is shown in Figure 5a,b regardless of the presence or absence of known disease genes. Previous studies [28] have already pointed out the problem of network propagation algorithms that are sensitive to gene degrees in the PPI network.

HerGePred showed high prediction accuracy with known disease genes (Figure 5a), whereas it had low accuracy without known disease genes (Figure 5b). HerGePred reconstructs a heterogeneous network using a graph embedding technique such as node2vec. This method measures the similarity of nodes based on structural equivalence, which represents the structural roles of nodes such as hubs and bridges. This topological analysis might be more susceptible to the prediction setting without known disease genes because all links to genes are removed for each disease. The low accuracy of HerGePred appeared particularly in the prediction of genes in higher degrees, as shown in Figure 5b.

IDLP also had a lower accuracy for the prediction of genes in higher degrees, as shown in both Figure 5a,b. Because IDLP treated the gene and disease networks as variables, even if this algorithm was based on network propagation, it predicted low-degree genes relatively well. However, as the gene degree increased, the number of successful predictions generally decreased.

## 5. Conclusions

Identifying disease-gene associations is crucial for detecting disease-causing genes and understanding disease mechanisms. Network-based approaches have recently been highlighted for their efficient disease-gene association prediction. In this survey, we provide a comprehensive overview of the existing network-based methods in three categories as follows: methods using graph-theoretic algorithms such as random walk, propagation and path search; methods using machine learning algorithms such as deep learning; and integrative methods of graph theory and machine learning techniques. We compared the prediction performance of the six selected methods using a heterogeneous disease-gene network that was constructed by integrating a genome-wide PPI network, an ontology-based disease network from HPO, and known disease-gene associations from OMIM.

To demonstrate prediction performance of each method, we employed ROC and recall. For ROC, we used a threshold, called a ranking parameter in our experiments, which indicates how many disease-associated genes are predicted for each disease. The ROC then showed the changes of the true positive rate and false positive rate among the predicted results within the threshold. The recall values, correct predictions out of actual disease-gene associations, were achieved by varying the threshold. In our experiments, we did not show precision, correct predictions out of predicted results. Because all previous methods had very low precision in this genome-scale analysis, comparing precision was meaningless.

Our experimental results revealed that HerGePred, an integrative method of graph theory and machine learning techniques, outperformed the others overall. This method had the best performance in the presence of known disease-associated genes, i.e., when at least one link to a gene was provided for the query disease. Although it had the lowest prediction accuracy among the three selected methods in the absence of known disease genes, it predicted well disease-associated genes with low degrees in the PPI network. This method only had difficulty predicting the genes with high degrees when disease-related genes are unknown.

In contrast, PRINCE, which adopts a typical network propagation algorithm, had the best performance in the absence of known disease-associated genes whereas it was less competitive in the presence of known disease-associated genes, particularly when the rank parameter value was small. The prediction accuracy of PRINCE was also greatly influenced by the degree of disease genes in a PPI network. This means that the network propagation algorithm predicts well the links from the genes of higher degrees, which contain more information topologically and biologically.

Relatively, IDLP and HerGePred are less affected by the degree of disease genes in a PPI network. The strength of IDLP is to handle noise that may exist in the gene network and disease network. However, if very reliable network data are used, the IDLP algorithm may not be advantageous. In terms of elapsed time, PRINCE and RWR run very fast, whereas HerGePred and NetCore need very long time to complete prediction.

From our experimental results, we can conclude that integrative methods such as HerGePred and IDLP performed better than methods using graph theory only. Moreover, PRINCE, HerGePred and IDLP, which take a heterogeneous network as input, performed better than RWR, DADA, and NetCore, which use a homogeneous PPI network only. These results indicate that both PPIs and disease similarities undoubtedly contributed to improving the prediction performance. Consolidating all relevant data into a weighted heterogeneous network enables us to view the prediction problem at a single system level and devise systematic approaches more effectively using network features. As a future direction, we can address the need to integrate heterogeneous and multiplex networks for more effective use of diverse network features.

These studies for predicting disease-gene associations can be extended to a more challenging problem of drug repositioning. In the recent pandemic, our top priority was to quickly develop medication to treat the new disease. However, it usually takes too long to bring new drugs to the market. The costly and time-consuming paradigm of drug development is not suitable for handling rapidly emerging and widespread diseases. One of the best solutions is to choose some already approved drugs to control such diseases, called drug-repositioning, based on the assumption that approved drugs do not have any critical side-effects. Network-based computational approaches, similar to the methods introduced in this survey, can be applied to solve the problem of drug repositioning by predicting drug-target-disease associations.

## Figures and Tables

**Table 1:** List of network-based methods for disease-gene association prediction including data sources, input network type, and key techniques.

Method	Data Source	Network Format	Technique
RWR [25]	OMIM, HPRD, BIND, BioGrid, IntAct, STRING	homogeneous network	random walk
RWRH [26]	OMIM, HPRD, MimMiner	heterogeneous network	random walk
PRINCE [27]	OMIM, HPRD, GO	heterogeneous network	network propagation
DADA [28]	OMIM, HPRD, BIND, BioGrid, MimMiner	homogeneous network	random walk
RWR-MH [29]	OMIM, HPO, Interactome	heterogeneous, multiplex network	random walk
PhenoRank [30]	OMIM, HPRD, BioGrid, IntAct, HPO	heterogeneous network	network propagation
NetCore [31]	DisGeNet, ConsensusPathDB	homogeneous network	random walk
PRYNT [32]	CTD, STRING	homogeneous network	path search, random walk
CIPHER [33]	OMIM, HPRD, BIND, MimMiner	heterogeneous network	linear regression
CrossRank [34]	OMIM, PubMed	tissue-specific heterogeneous network	network propagation
pBRIT [35]	HPO, ConsensusPathDB, GO	homogeneous network	Bayesian ridge regression
Scuba [36]	HPRD, STRING, Reactome, PID	homogeneous network	graph node kernels
IDLP [37]	OMIM, HPRD, BioGrid, IntAct, Interactome, MimMiner	heterogeneous network	network propagation
HerGePred [38]	HPO, DisGeNet, MalaCard, Orphanet	heterogeneous network	network embedding, random walk

**Table 2:** Numbers of nodes and edges in each network for the experiment.

Experimental Data	Number of Nodes	Number of Edges
Gene network	14,663	258,476
Disease network	6465	4,354,956
Disease-gene associations	-	5024

**Table 3:** AUC results evaluated by gene ranks for disease-gene association prediction with known disease genes from sample-1.

Method	*r* = 100	500	1000	5000	10,000
RWR	0.813	0.800	0.791	0.775	0.733
PRINCE	0.561	0.636	0.620	0.694	0.703
DADA	0.816	0.789	0.785	0.782	0.741
IDLP	0.596	0.559	0.524	0.650	0.791
HerGePred	0.838	0.863	0.835	0.800	0.778
NetCore	0.790	0.805	0.792	0.764	0.745

**Table 4:** Recall values evaluated by gene ranks for disease-gene association prediction with known disease genes from sample-1.

Method	*r* = 100	500	1000	5000	10,000
RWR	0.225	0.350	0.425	0.657	0.863
PRINCE	0.077	0.197	0.306	0.673	0.898
DADA	0.230	0.357	0.436	0.680	0.889
IDLP	0.019	0.091	0.176	0.617	0.661
HerGePred	0.302	0.401	0.471	0.719	0.889
NetCore	0.197	0.295	0.364	0.633	0.817

**Table 5:** AUC results evaluated by prediction scores for disease-gene association prediction with known disease genes from sample-1.

Method	*s*/*n* = 100	500	1000	5000	10,000
RWR	0.391	0.533	0.443	0.528	0.550
PRINCE	0.543	0.643	0.620	0.694	0.702
DADA	0.442	0.682	0.540	0.548	0.484
IDLP	0.663	0.532	0.543	0.709	0.632
HerGePred	0.795	0.834	0.835	0.794	0.774
NetCore	0.622	0.738	0.764	0.761	0.732

**Table 6:** Recall values evaluated by prediction scores for disease-gene association prediction with known disease genes from sample-1.

Method	*s*/*n* = 100	500	1000	5000	10,000
RWR	0.007	0.037	0.104	0.439	0.745
PRINCE	0.079	0.195	0.306	0.673	0.898
DADA	0.012	0.030	0.058	0.209	0.715
IDLP	0.016	0.095	0.176	0.357	0.552
HerGePred	0.302	0.411	0.469	0.722	0.889
NetCore	0.179	0.325	0.394	0.638	0.826

**Table 7:** AUC results evaluated by gene ranks for disease-gene association prediction without known disease genes from sample-2.

Method	*r* = 100	500	1000	5000	10,000
PRINCE	0.485	0.655	0.576	0.685	0.663
IDLP	0.333	0.478	0.559	0.698	0.644
HerGePred	0.257	0.647	0.536	0.534	0.520

**Table 8:** Recall values evaluated by gene ranks for disease-gene association prediction without known disease genes from sample-2.

Method	*r* = 100	500	1000	5000	10,000
PRINCE	0.086	0.171	0.303	0.594	0.846
IDLP	0.011	0.086	0.166	0.446	0.749
HerGePred	0.011	0.034	0.069	0.343	0.657

**Table 9:** AUC results evaluated by prediction scores for disease-gene association prediction without known disease genes from sample-2.

Method	*s*/*m* = 100	500	1000	5000	10,000
PRINCE	0.480	0.651	0.571	0.684	0.663
IDLP	0.550	0.367	0.468	0.642	0.657
HerGePred	0.351	0.566	0.567	0.499	0.531

**Table 10:** Recall values evaluated by prediction scores for disease-gene association prediction without known disease genes from sample-2.

Method	*s*/*m* = 100	500	1000	5000	10,000
PRINCE	0.086	0.171	0.303	0.594	0.846
IDLP	0.011	0.097	0.194	0.451	0.640
HerGePred	0.023	0.040	0.074	0.371	0.646

## Abbreviations

The following abbreviations are used in this manuscript:

PPI	Protein-Protein Interaction
GWAS	Genome-wide association studies
GO	Gene Ontology
HPO	Human Phenotype Ontology
ROC	Receiver operating characteristic
AUC	The area under the curve
